# Seroprevalence of Swine Influenza A Virus (swIAV) in Commercial Pig Farms in Greece (2023–2026): Age, Regional and Temporal Patterns

**DOI:** 10.3390/vetsci13101041

**Published:** 2026-10-02

**Authors:** Vasileios G. Papatsiros, Georgios I. Papakonstantinou, Constantinos S. Kyriakis, Dimitrios C. Sakkas, Zoi Prentza, Ilias Terzidis, Konstantinos Koutoulis, Charalambos Billinis

**Affiliations:** 1 Clinic of Medicine, Faculty of Veterinary Medicine, University of Thessaly, 43100 Karditsa, Greece; geopapak@vet.uth.gr; 2 Department of Pathobiology, College of Veterinary Medicine, Auburn University, Auburn, AL 36849, USA; csk0021@auburn.edu (C.S.K.); dzs0140@auburn.edu (D.C.S.); 3 Department of Poultry Diseases, Faculty of Veterinary Medicine, University of Thessaly, 43100 Karditsa, Greece; zoiprentza@hotmail.com (Z.P.); kkoutoulis@uth.gr (K.K.); 4 Swine Technical Support, Hipra Hellas SA, 10441 Athens, Greece; ilias.terzidis@hipra.com; 5 Department of Microbiology and Parasitology, Faculty of Veterinary Medicine, University of Thessaly, 43100 Karditsa, Greece; cbillinis@uth.gr

**Keywords:** swine influenza, swIAV, seroprevalence, pigs, Greece, zoonosis, maternally derived antibodies

## Abstract

Swine influenza A virus (swIAV) is an endemic respiratory pathogen of pigs with zoonotic relevance. We analysed retrospective serological data from routine diagnostic submissions (January 2023–May 2026) to a single laboratory, comprising 2003 sera from 66 reports on 44 commercial farrow-to-finish farms in seven regions of mainland Greece, tested with a nucleoprotein ELISA that cannot distinguish vaccine-, maternally, or infection-derived antibodies. Overall seroprevalence was 46.4% (929/2003; farm-clustered 95% CI 39.1–53.8%); at least one seropositive animal was found in 51 of 61 farm-years and in 36 of 44 farms. Seroprevalence differed by production stage (*p* < 0.001): it was highest in multiparous sows (75.9%) and suckling piglets (70.7%), both likely influenced by sow vaccination and maternally derived antibodies, intermediate in gilts (46.0%) and nursery pigs (38.6%), and lowest in growers (24.8%) and finishers (24.0%), in which antibodies indicate prior exposure rather than current circulation. No consistent temporal trend was detected, although some adjusted models suggested higher seroprevalence in 2025–2026. Regional seroprevalence ranged from 10.1% (Thessaly) to 61.0% (West Greece); the association was significant with the standard but not the bias-reduced cluster-robust estimator (*p* = 0.014 and *p* = 0.39) and persisted after adjustment for year and stage, but it rests on few farms per region and is considered exploratory. As a convenience sample of diagnostic submissions, these data provide descriptive rather than nationally representative estimates and support the addition of molecular, subtype-level surveillance.

## Simple Summary

Swine influenza is a common respiratory infection of pigs that can occasionally infect people, so understanding how prevalent it is on farms matters for both animal and public health. We analysed blood-test results from more than 2000 pigs on 44 commercial farms in seven regions of mainland Greece, submitted to a diagnostic laboratory between 2023 and 2026. About 46 in every 100 pigs had antibodies indicating past contact with the virus. Antibodies were most common in sows and suckling piglets, most likely because sows are vaccinated and pass antibodies to their piglets, and least common in unvaccinated growing and finishing pigs, in which antibodies indicate past exposure rather than proving that the virus was circulating at sampling. Levels did not change consistently over time, and regional differences require confirmation because several regions had few farms. Because the farms were not randomly selected, these figures are descriptive rather than national estimates; they support adding virus-detection testing to future surveillance.

## 1. Introduction

Global pig production has expanded substantially over recent decades, and intensification—larger herd sizes, higher stocking densities and increasingly integrated production chains—has become the prevailing model in most major pig-producing regions, including Southern Europe [1]. While intensification has driven gains in production efficiency, it has simultaneously created conditions that favour the introduction, amplification and endemic persistence of infectious pathogens, making systematic disease surveillance an increasingly indispensable component of modern swine health management.

Among the pathogens routinely monitored in commercial pig herds, swine influenza A virus (swIAV) occupies a distinctive position because its significance extends well beyond direct production losses. swIAV is one of the most important viral pathogens endemic in swine, and a major component of the porcine respiratory disease complex (PRDC). Clinically, swIAV causes an acute respiratory syndrome (fever, anorexia, coughing, dyspnoea) with high morbidity but generally low mortality. As a core PRDC component, it commonly predisposes to secondary co-infections and causes measurable production losses through reduced growth, poorer feed conversion and reproductive disturbances [2,3]. Recent systematic-review evidence indicates that endemic PRDC-associated respiratory disease imposes substantial economic losses, estimated at approximately €1.70–8.90 per nursery pig and €2.30–15.35 per fattening pig, in addition to reduced sow productivity, underscoring the practical relevance of accurate seroprevalence surveillance [4]. Importantly, swIAV is intrinsically zoonotic and of pandemic concern, given the established capacity of pigs to act as a “mixing vessel” in which avian-, human- and swine-adapted influenza A virus lineages can co-infect a single host and reassort, potentially generating novel viruses with pandemic potential.

This mixing-vessel capacity is attributed to the co-expression of both avian-type (α2,3-linked) and human-type (α2,6-linked) sialic acid receptors in the porcine respiratory tract [5] and has contributed to the emergence of the 2009 pandemic H1N1 virus, which arose from a multi-way reassortment of gene segments previously circulating in swine [6]. Surveillance of swIAV in pigs is coordinated internationally through structured networks such as the European Surveillance Network for Influenza in Pigs (ESNIP), whose most recent phase (ESNIP3, 2010–2013) [7] documented influenza A virus detection in approximately 31% of more than 9000 herds examined across 17 European countries, with substantial country-to-country variation in both detection frequency and circulating viral lineages and subtypes, including H1N1, H3N2 and H1N2 [7]. A recent Greek nationwide study using the same CIVTEST SUIS INFLUENZA ELISA kit employed in the present work reported swine influenza seroprevalence of 54% in vaccinated and 23% in unvaccinated commercial farrow-to-finish farms across the country [8], providing a methodologically consistent reference point for the present analysis. In Greece specifically, serological evidence of infection with the 2009 pandemic H1N1 lineage has been documented in swine following its emergence [9], consistent with the broader European pattern of multiple co-circulating swIAV lineages (H1N1, H3N2 and H1N2) [7]; lineage-level characterization of currently circulating strains was, however, outside the scope of the present serological survey.

Because highly pathogenic avian influenza (HPAI) also circulates recurrently in Greek wild birds and, on occasion, poultry [10,11], swine influenza surveillance is additionally relevant for the wider livestock sector; however, this study did not include anti-H5 haemagglutinin serology and therefore cannot directly address avian-to-swine exposure.

The present study analysed swIAV serological data from 44 commercial farrow-to-finish pig farms (of the 46 farms in the diagnostic dataset) distributed across seven administrative regions of mainland Greece. We report (i) overall animal-level seroprevalence and event- and farm-level positivity, (ii) temporal variation across 4 sampling years (2023–2026), (iii) variation across six standardized age/production stages, and (iv) regional heterogeneity across seven administrative regions of mainland Greece. Compared with the earlier Greek survey [8], which covered farms sampled between March 2019 and April 2023, the present study extends the observation period to 2023–2026, covers seven rather than three geographical areas, applies a standardized six-stage age/production classification and explicitly accounts for farm-level clustering in all comparisons. Unlike [8], in which blood samples were collected specifically for research by stratified random sampling in farms without clinical respiratory signs, the present data derive from routine diagnostic submissions; the two datasets therefore differ in sampling design, and their temporal overlap is limited to January–April 2023 (Section 4.1).

## 2. Materials and Methods

### 2.1. Study Design and Data Source

This was a retrospective, observational, multi-regional cross-sectional study based on routinely collected veterinary diagnostic data. The dataset comprised 78 farm-sampling events (laboratory reports) from 46 individually identified commercial farrow-to-finish pig farms across mainland Greece, drawn from diagnostic-laboratory reports issued between January 2023 and May 2026 by a single commercial veterinary diagnostic laboratory. Each farm-sampling event was treated as a cross-sectional observation, with repeated events from the same farm modelled as correlated observations (Section 2.5). The investigators applied no experimental intervention; all sampling, testing and vaccination decisions reflected routine clinical and herd-health management decisions made independently by participating farms and their attending veterinarians. Farms were eligible for inclusion if they submitted serum samples for routine swIAV ELISA testing to the laboratory during the study period and returned valid results for at least one animal; no farm was excluded based on size, health status, production type or region, and no sampling protocol was imposed by the investigators. Because inclusion was contingent on voluntary diagnostic submission to a single commercial laboratory rather than on random selection, the sample is subject to selection bias and should be extrapolated to the national swine population only with caution (Section 6). The reason for submission (e.g., investigation of clinical respiratory disease versus routine herd-health monitoring) was not recorded in the diagnostic dataset available for analysis, so results could not be stratified by submission motive; the estimates presented here are therefore laboratory-submission-based descriptive estimates and should not be used as a national baseline. Of the 78 laboratory reports (farm-sampling events), 66, from 44 unique farms, included swIAV serology with at least one valid serum result; the remaining 12 reports did not include swIAV testing and contributed no data to the present analysis. For herd-level summaries, reports from the same farm within the same calendar year (four farm-years comprising nine reports) were combined, yielding 61 farm-year sampling units. Of the 2003 valid swIAV results, 1989 (927 positive) were assigned to one of the six standardized stages; 14 samples, all collected in 2023, could not be assigned (12 samples without an age label, all negative, and 2 samples from boars, both positive) and were excluded from the age-stratified analysis only.

### 2.2. Farms

The 46 participating farms were located across 7 administrative regions of mainland Greece, with herd sizes ranging from 100 to 1200 sows per farm (22,920 sows in total). Table 1 summarizes the regional distribution of the participating farms and their breeding sow capacity.

### 2.3. Serological Testing

Individual serum samples were tested for swIAV antibodies using a commercial indirect ELISA (CIVTEST SUIS INFLUENZA^®^; HIPRA, Amer, Spain), with results classified as negative (≤20) or positive (>20) according to the manufacturer-declared sample-to-positive cut-off recorded in the original laboratory reports; this threshold was not modified for the study. This assay detects antibodies against the influenza A nucleoprotein (NP), a conserved internal antigen shared by all subtypes; consequently, it is not subtype-specific and cannot distinguish vaccine-induced from naturally acquired antibody responses. Samples were collected from small groups of animals (typically 3–5, occasionally up to 20 per age/parity stratum, with several strata usually sampled at each event, so that the total number per event varied; Section 3.5) spanning suckling piglets through nursery, growing and finishing pigs to nulliparous gilts and multiparous sows. Although the original diagnostic submissions occasionally also included urine or oral fluid samples, these were not tested by the swIAV ELISA and were not included; only serum samples were analysed in the present study. A standardized post hoc classification scheme was applied to harmonize age/parity terminology across farms and sampling occasions into six standardized stages: suckling piglets (≤3 weeks), nursery/weaners (4–8 weeks), growers (9–16 weeks), finishers (>16 weeks), gilts/nulliparous sows, and multiparous sows. Molecular (oral fluid-based) swIAV data reported by the source laboratory fell outside the blood-only inclusion criterion adopted for this dataset and are therefore not included in the present analysis; the absence of any molecular or subtyping data is a defining limitation of this study (Section 6).

### 2.4. Vaccination Context

Across all 46 participating farms, breeding sows were routinely vaccinated against swIAV as part of the standard herd-level vaccination protocol, alongside porcine reproductive and respiratory syndrome virus (PRRSV), Aujeszky’s disease (gE-deleted marker vaccine), *Erysipelothrix rhusiopathiae* and porcine parvovirus. Piglets, growers and finishers were not included in the standard swIAV vaccination protocol reported for this farm sample. Consequently, the high sow seroprevalence documented below reflects, to a substantial and likely dominant degree, a combination of vaccine-induced humoral immunity and cumulative natural exposure across multiple reproductive cycles that cannot be fully disentangled with the available data, whereas seropositivity in unvaccinated growing and finishing pigs is more likely to reflect prior natural exposure, although residual MDA (particularly in younger animals), antibodies persisting after earlier infection and non-specific assay reactions cannot be excluded. Vaccination information was available only at the farm (protocol) level; individual vaccination status, vaccine product and timing were not recorded.

### 2.5. Statistical Analysis

**Figure 1:**
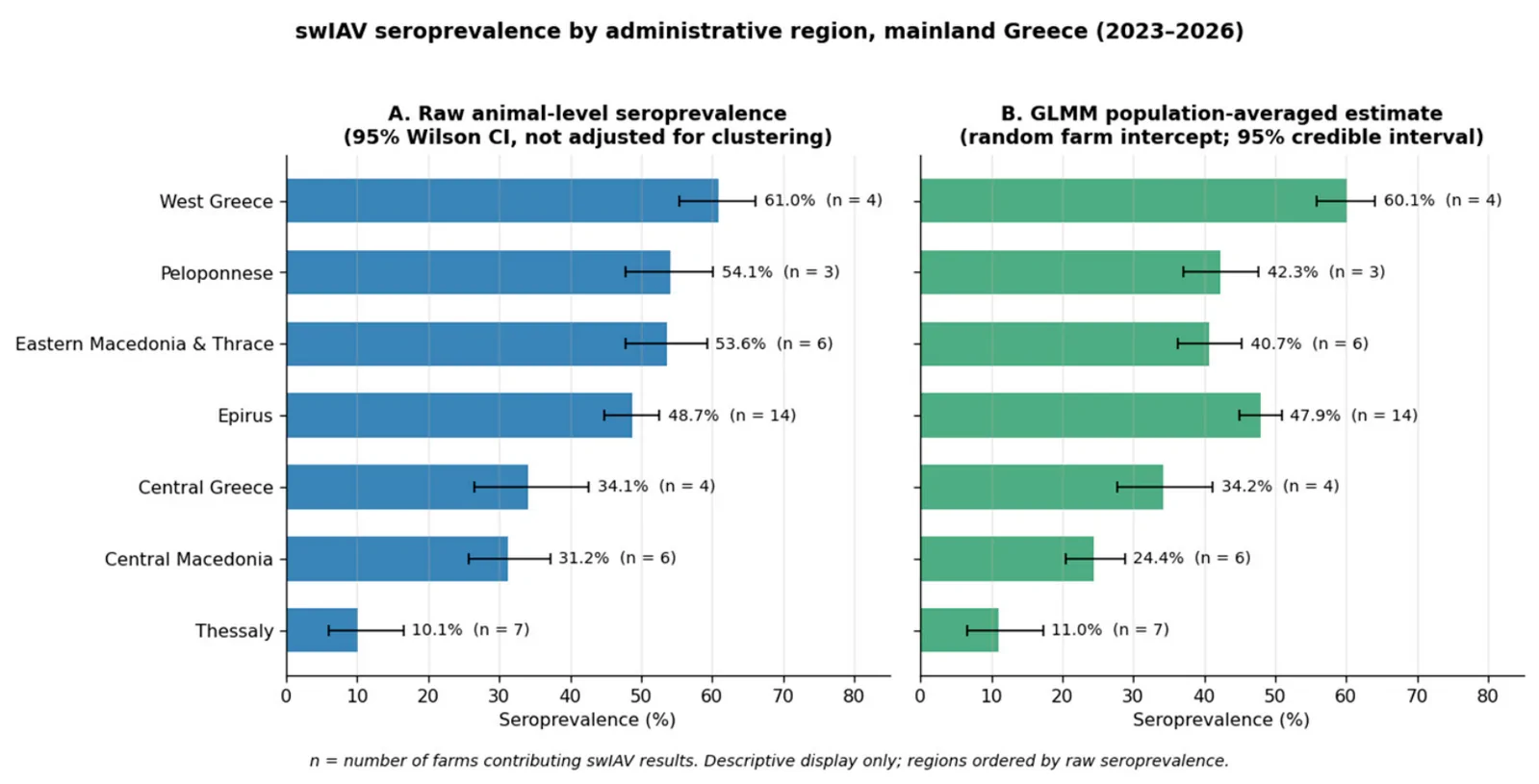
Regional distribution of swIAV seroprevalence across the 7 administrative regions of mainland Greece during 2023–2026. (**A**) Raw animal-level seroprevalence (%), calculated as the proportion of seropositive animals among all tested animals within each region. Error bars show 95% Wilson confidence intervals, which do not account for within-farm clustering; *n* indicates the number of farms contributing swIAV results. (**B**) Regional seroprevalence estimates derived from a generalized linear mixed model (GLMM) with a random farm effect, showing population-averaged estimates with 95% credible intervals, in which heavily sampled farms do not dominate their region’s estimate (Table 5) (descriptive display only; not used for inference). Higher seroprevalence was observed in West Greece, whereas Thessaly showed the lowest seroprevalence across the study period; raw estimates, not adjusted for age structure or sampling period; regional differences are exploratory.

Seroprevalence was calculated as the proportion of positive individual samples among valid tests, with 95% confidence intervals estimated using the Wilson score method [12], which performs substantially better than the Wald interval for small samples and for proportions close to 0% or 100%. These Wilson intervals are descriptive, individual-level intervals that do not account for within-farm clustering; for the overall estimate and for each stratum in Table 2, Table 3 and Table 4, farm-clustered 95% CIs were additionally obtained from intercept-only GEE models with the bias-reduced covariance estimator. Herd-level positivity (at least one seropositive animal) was summarized at three levels: per laboratory report (sampling event), per farm-year sampling unit (reports from the same farm within the same calendar year combined; the unit underlying the 83.6% figure) and per unique farm (at least one seropositive animal in any report); because farms could contribute more than one report or year, these measures are reported separately. To describe within-unit heterogeneity, the distribution of the within-farm-year proportion of seropositive animals was also summarized. Because individual animals were sampled within farms rather than independently, within-farm correlation of serological status was formally assessed using the intraclass correlation coefficient (ICC) via one-way random-effects ANOVA, and the corresponding design effect (DEFF = 1 + ($\bar{m}$ − 1) × ICC, where $\bar{m}$ is the mean number of animals per farm, the clustering unit) and effective sample size were derived. Because the number of valid samples per event varied, the distribution of cluster sizes (median, range and coefficient of variation, CV) is reported, and a design effect accounting for unequal cluster sizes, DEFF = 1 + [(1 + CV^2^) × $\bar{m}$ − 1] × ICC [13], was also calculated. To address this clustering, comparisons across sampling year, age/production stage and administrative region were assessed using farm-clustered generalized estimating equations (GEEs) [14] with a logit link, binomial family, independence working-correlation structure (selected by QIC; see below) and canonical farm identity as the clustering unit, yielding cluster-robust (sandwich) standard errors; joint significance was assessed with a robust Wald test, and, for the principal pairwise contrasts, odds ratios (ORs) with 95% confidence intervals are reported alongside *p*-values. The contrast between multiparous sows and finishers was designated as the principal contrast on biological grounds, because it compares the stage most exposed to vaccination with the unvaccinated stage least likely to carry MDA; because this contrast was not formally pre-registered, all 15 pairwise stage contrasts are additionally reported with Benjamini–Hochberg adjustment (Supplementary Table S4). Given the modest number of farm clusters available (44–46), all GEE models were additionally refitted using the small-sample bias-reduced (Mancl–DeRouen) sandwich covariance estimator [15], which corrects the anti-conservative bias of the standard robust estimator when the cluster count is below approximately 40–50; where the two estimators disagreed on statistical significance, the more conservative bias-reduced result was treated as primary. Benjamini–Hochberg false-discovery-rate (FDR) correction [16] was applied across the three stratification comparisons reported in this manuscript (year, stage and region). The FDR family therefore comprised exactly three tests (year, stage and region), and the correction was applied separately to the standard and bias-reduced covariance estimators; all FDR-adjusted *p*-values reported in this manuscript refer to this three-test family, and the corresponding raw and adjusted *p*-values are listed in Supplementary Table S3. The linear year-trend test was reported separately and was not part of this family. A GEE served as the primary, population-averaged framework for between-stratum hypothesis testing, chosen for its robustness to misspecification of the working-correlation structure, including all inference on regional differences; the GLMM had no inferential role and was used only descriptively, as a subject-specific, partial-pooling model, to display shrinkage estimates for sparsely sampled regions (Table 5, Figure 1B). The GLMM was a binomial mixed logistic model with region as a fixed effect and a normally distributed random intercept for farm, fitted by variational Bayes using the BinomialBayesMixedGLM class of the Python statsmodels library (version 0.15; fit_vb method with default priors). Region-specific population-averaged seroprevalences were obtained by integrating the inverse logit of each regional linear predictor over the estimated normal distribution of farm random effects (standard deviation 1.36 on the logit scale; 60-point Gauss–Hermite quadrature), so that each farm contributes more evenly than in the pooled raw proportion and heavily sampled farms do not dominate their region’s estimate. Further, 95% credible intervals were obtained by propagating 20,000 draws from the approximate posterior distribution of each regional fixed effect through the same integration; because variational Bayes tends to underestimate posterior uncertainty, these intervals should be regarded as approximate. Because the 66 swIAV reports derived from 44 farms, several farms contributed more than one report, in some cases in different years; farm identity was therefore used as the clustering unit so that repeated observations from the same farm, including across years, were treated as correlated (an explicit within-farm autoregressive temporal structure was not separately estimated given the few repeated-year observations per farm, and a sensitivity analysis excluding the two most frequently resampled farms, for the overall, regional and stage-specific estimates, is reported in Section 3.5). The binomial GEE specification was checked for overdispersion using the Pearson-based dispersion statistic, and the fit of alternative working-correlation structures (exchangeable versus independence) was examined using the QIC criterion [17]. QIC favoured the independence working correlation for all three stratification models (year 2765 vs. 2856; stage 2401 vs. 2436; region 2642 vs. 2679, independence vs. exchangeable), which was therefore used for all reported GEE models; models with exchangeable working correlation are reported as a sensitivity analysis. Because sampling in 2026 covered January–May only, 2026 was treated as a partial year; the monthly distribution of samples by year was tabulated, and a sensitivity analysis of the year effect restricted to samples collected in January–May of each year was performed. To explore confounding of the regional comparison by age structure and sampling period, the region × year and region × stage distributions of samples were tabulated (Supplementary Table S2), the full multivariable GEE (year + stage + region) was fitted, and a reduced multivariable GEE with collapsed categories (year: 2023–2024 vs. 2025–2026; stage: suckling/nursery, grower/finisher, breeding animals) and a Firth penalized logistic regression (ignoring clustering) were fitted as sensitivity analyses (Supplementary Table S6). Serological records with missing, invalid or indeterminate ELISA results were excluded listwise from the relevant denominators, records lacking age/parity information were excluded from the age-stratified analysis only, and no imputation was performed, so that all reported proportions refer to valid test results only.

## 3. Results

### 3.1. Overall and Herd-Level (Report, Farm-Year and Farm) Seroprevalence

Of 2003 individual serum samples tested for swIAV across the four-year study period, 929 were positive, yielding an overall seroprevalence of 46.4% (95% Wilson score CI 44.2–48.6%); farm-clustered GEE 95% CI 39.1–53.8%, bias-reduced 38.8–54.1%. At least one seropositive animal was detected in 51 of 61 farm-year sampling units (83.6%), in 56 of 66 individual laboratory reports (84.8%) and in 36 of 44 unique farms (81.8%). Because each farm-year unit included on average about 33 animals, a high herd-level positivity is almost automatically expected when individual seroprevalence is close to 46%, so these figures add little information by themselves. More informative are the distribution of the within-farm-year proportion of seropositive animals (median 42.1%, interquartile range 12.5–60.0%, range 0–100%) and the finding that 10 of the 61 farm-years (16.4%) had no seropositive animal, although 3–34 animals (median 18.5) were tested in each; for the larger of these units (≥20 animals) such an outcome would be highly improbable under a random, non-clustered distribution of seropositivity (0.536^20^ ≈ 4 × 10^−6^), indicating marked between-farm heterogeneity, consistent with the ICC reported in Section 3.5.

### 3.2. Temporal Trend (2023–2026)

Table 2 presents swIAV seroprevalence by sampling year. The crude, unclustered chi-squared test suggested statistically significant year-to-year variation (*p* < 0.001); however, once farm-level clustering was accounted for using farm-clustered GEEs, the categorical year effect was not statistically significant (*p* = 0.50), nor was the linear-trend test (OR = 1.12 per year, 95% CI 0.90–1.40; *p* = 0.29). Refitting with the small-sample bias-reduced covariance estimator yielded uniformly larger *p*-values, reinforcing that no genuine temporal trend in swIAV seroprevalence was detected over the study period once clustering was appropriately accounted for. Given only four sampling years, the trend analysis has limited power; based on the standard error of the farm-clustered trend estimate, the minimum detectable linear trend (80% power, two-sided α = 0.05) was an OR of approximately 1.37 per year (1.42 with the bias-reduced estimator), i.e., an increase of about 8–9 percentage points per year around the observed mean, so smaller trends cannot be excluded, and this null result should not be over-interpreted. The 2026 data cover January–May only, so 2026 is a partial year; the numbers of events and farms per year are given in the footnote to Table 2 and the monthly distribution of samples in Supplementary Table S1. In a sensitivity analysis restricted to samples collected in January–May of each year (1445 samples), seroprevalence was 43.4% (171/394) in 2023, 31.8% (56/176) in 2024, 53.1% (255/480) in 2025 and 45.8% (181/395) in 2026, with no significant year effect (farm-clustered GEE *p* = 0.086; bias-reduced *p* = 0.16) or linear trend (OR = 1.10 per year, 95% CI 0.86–1.40; *p* = 0.46). However, with the exchangeable working correlation, which weights within-farm comparisons more heavily, the year effect was significant (bias-reduced *p* < 0.001; linear-trend OR = 1.26 per year, 95% CI 1.12–1.42), indicating that among repeatedly sampled farms seroprevalence tended to be higher in later years; the absence of a temporal trend is therefore not robust to the choice of working correlation and should be interpreted with caution. In the adjusted models (Section 3.4), the year effect was significant with the standard estimator (*p* = 0.027) but not with the bias-reduced estimator (*p* = 0.11), and the reduced model suggested higher seroprevalence in 2025–2026 than in 2023–2024 (OR 1.88, 95% CI 1.11–3.19). Several farms were sampled in more than one year; because these repeated observations were modelled as correlated within farm, they do not inflate the precision of the year comparison, but year-to-year differences may partly reflect changes in which farms were sampled rather than true temporal change.

### 3.3. Seroprevalence by Age/Production Stage

Stage information was available for 1989 of the 2003 samples (927 positive); 14 samples from 2023 (12 without an age label and two from boars; two positive) were excluded from this analysis only. Both crude chi-squared tests and farm-clustered, FDR-corrected GEE Wald tests revealed highly significant differences in swIAV seroprevalence across age/production stages (cluster-adjusted *p* < 0.001), a result robust to farm-level clustering. Seroprevalence was highest in suckling piglets (70.7%) (*n* = 198, drawn from 23 contributing farms) and multiparous sows (75.9%), intermediate in gilts/nulliparous sows (46.0%) and nursery/weaner pigs (38.6%), and lowest in growers and finishers (24.8% and 24.0%, respectively; Table 3), describing a trough-shaped rather than simple monotonic pattern. In the principal pairwise contrast (selected on biological grounds; Section 2.5), extracted from the same farm-clustered, bias-reduced GEE model used throughout this study, multiparous sows were substantially more likely to be seropositive than finishers (OR = 10.0, 95% CI 3.7–26.7). Of all 15 pairwise contrasts (Supplementary Table S4), 11 remained significant after FDR adjustment with the bias-reduced estimator, the exceptions being suckling piglets vs. multiparous sows, nursery/weaners vs. gilts, nursery/weaners vs. finishers and growers vs. finishers. Interpretation of both peaks must account for vaccination: breeding sows on all farms were routinely swIAV-vaccinated, and the high seroprevalence in suckling piglets is most plausibly explained by maternally derived antibody (MDA) transferred from these vaccinated dams via colostrum, although this cannot be confirmed with cross-sectional serology alone, whereas growing/finishing pigs were not part of the vaccination protocol (Section 2.4). Among the unvaccinated stages (nursery through finisher), seroprevalence declined from 38.6% in nursery/weaners to approximately 24% in growers/finishers (growers 24.8%, finishers 24.0%). Because nursery/weaners were defined as 4–8 weeks of age, and swIAV MDA from vaccinated dams may persist for several weeks after weaning [18], an unknown part of the 38.6% in this stage may represent residual MDA rather than active immune responses; no age-specific antibody titres or MDA decay data were available to quantify this. The approximately 24% seropositivity in growers/finishers may be consistent with limited natural exposure during the growing period, but alternative explanations cannot be excluded, including antibodies persisting after earlier infection, residual MDA in the youngest growers, and non-specific or cross-reactive ELISA reactions. Moreover, seropositivity indicates prior exposure and does not by itself demonstrate active viral circulation at the time of sampling. The subsequent rise in breeding animals is best interpreted as a mixture of repeated vaccinations and cumulative exposure over the years that the present data cannot disentangle. The age curve is therefore described here only descriptively—high in suckling piglets, lower in nursery pigs, lowest in growers/finishers and high again in breeding animals; the proposed mechanisms (MDA decline, limited exposure during growing, and vaccination/cumulative exposure in breeders) remain hypotheses that the present data cannot confirm, and both peaks (suckling piglets and multiparous sows) are likely to be vaccine-context-dependent rather than markers of natural viral exposure.

**Figure 2:**
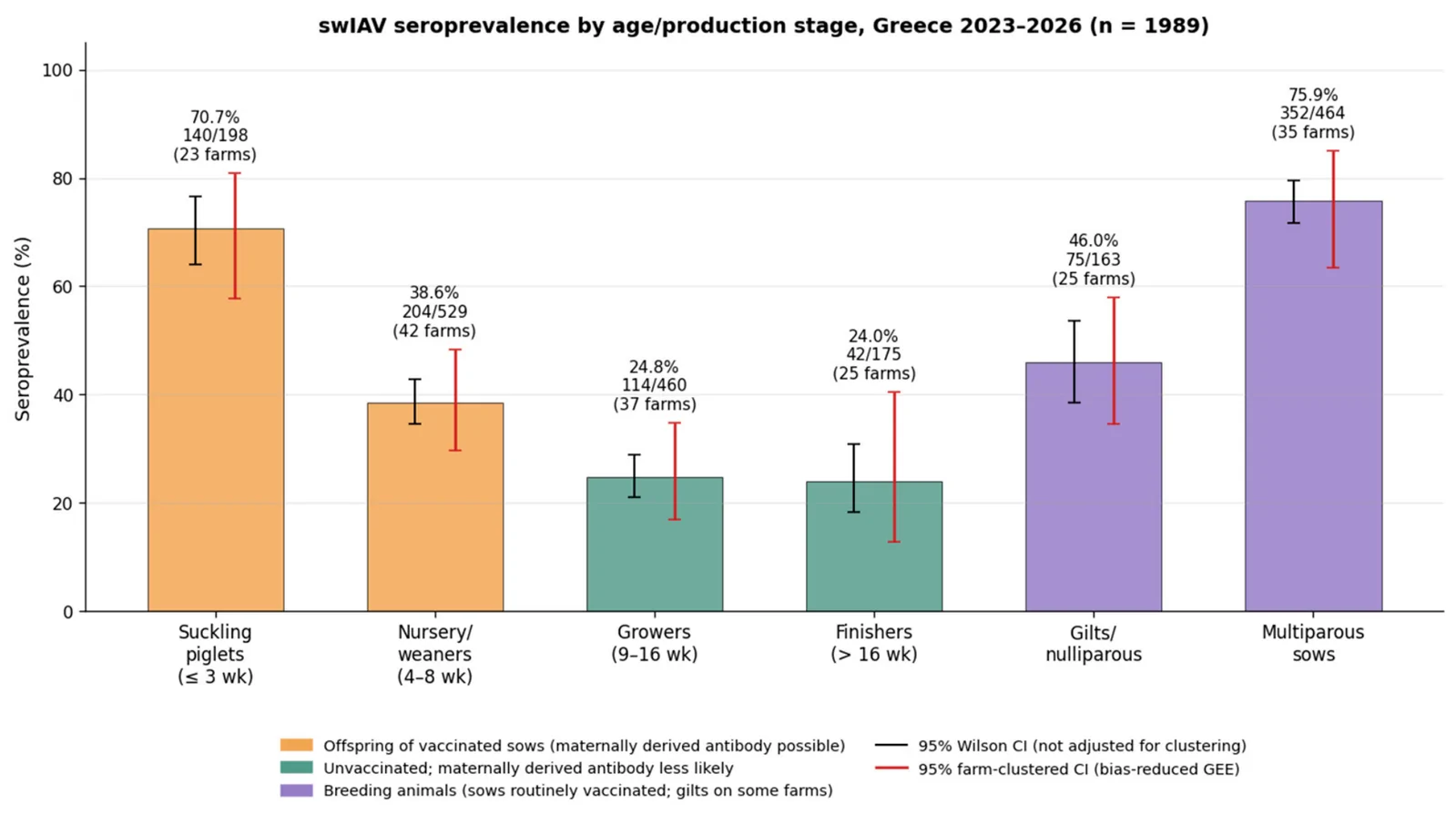
Observed swine influenza A virus (swIAV) seroprevalence by age/production stage in commercial pig farms in Greece (2023–2026). Seroprevalence was highest in suckling piglets (70.7%), most likely reflecting maternally derived antibodies (MDAs) acquired through colostrum from vaccinated sows, and in multiparous sows (75.9%), likely reflecting routine vaccination and cumulative lifetime exposure. Bars show animal-level seroprevalence; black error bars are 95% Wilson CIs (not adjusted for clustering) and red error bars are 95% farm-clustered CIs (bias-reduced GEE); labels give *n* positive/*n* tested and the number of contributing farms, and colours indicate vaccination context.

Figure 2 shows the observed seroprevalence by age/production stage with unadjusted and farm-clustered 95% CIs, grouped by vaccination context, distinguishing the two peaks likely influenced by vaccination or MDA from the intervening trough among the unvaccinated production stages.

### 3.4. Regional Analysis

SwIAV seroprevalence varied across the seven administrative regions represented in the dataset (Table 4), ranging from 10.1% in Thessaly to 61.0% in West Greece (OR West Greece vs. Thessaly = 13.9, 95% CI 2.6–74.1 under standard robust variance, extracted from the full seven-region model). The crude chi-squared test was statistically significant (*p* < 0.001), and this regional association remained significant after farm-clustered GEE testing with standard robust variance and FDR correction (*p* = 0.014). However, refitting with the small-sample bias-reduced (Mancl–DeRouen) covariance estimator reduced this to non-significance (bias-reduced FDR *p* = 0.39). The multivariable, mutually adjusted GEE model (seropositivity ~ year + stage + region) failed to converge for swIAV under the exchangeable working correlation owing to sparse cells once all three factors were modelled jointly. Because regions differed in the age/production stages and years sampled (Supplementary Table S2)—for example, Central Greece was sampled only in 2024 and 2026, and Peloponnese and Central Macedonia not at all in 2024—and vaccination was documented only at protocol level, the unadjusted regional differences may be partly confounded by age-group composition, sampling period and farm-level management. Under the independence working correlation used throughout (Section 2.5), the fully adjusted model converged: the regional effect persisted after adjustment for year and stage (standard robust *p* < 0.001; bias-reduced *p* = 0.037), as did stage (*p* < 0.001), whereas the adjusted year effect was significant only with the standard estimator (*p* = 0.027; bias-reduced *p* = 0.11). A reduced model with collapsed year and stage categories gave similar results (region, bias-reduced *p* = 0.048), and the Firth penalized regression, which ignores clustering and is therefore anti-conservative, also indicated a regional effect (likelihood-ratio *p* < 0.001; Supplementary Table S6). Thessaly had low seroprevalence in every year sampled (0–25%). Adjusted ORs relative to Thessaly were, however, very imprecise (e.g., West Greece OR = 23.0, 95% CI 2.8–190), reflecting the small number of farms per region. When regions represented by fewer than five farms were merged with an adjacent region (Epirus with West Greece; Central Greece with Peloponnese), the pattern was unchanged, with seroprevalence ranging from 10.1% in Thessaly to 53.6% in Eastern Macedonia and Thrace and 52.7% in Western Greece (region effect: unadjusted *p* = 0.028, bias-reduced *p* = 0.17; adjusted for year and stage *p* < 0.001, bias-reduced *p* = 0.028; Supplementary Table S7). Several regions are represented by very few farms (Table 1: Peloponnese *n* = 3, West Greece *n* = 4), so the corresponding point estimates are unstable and are shown for completeness only; they should not be interpreted as precise regional prevalences. Accordingly, the regional gradient is best regarded as hypothesis-generating rather than confirmed. The multivariable model was specified as a farm-clustered binomial GEE with logit link and exchangeable working correlation, regressing seropositivity simultaneously on year, stage and region; non-convergence took the form of a non-positive-definite working-covariance matrix under this fully parameterized specification, reflecting the sparse and partially empty cells produced by the full cross-classification. The partial-pooling GLMM (random farm intercept; Table 5) was not used to replace the multivariable model and has no inferential role; it is presented only as a descriptive display of shrinkage estimates for the sparsely sampled regions.

As a descriptive complement (not used for inference) addressing the sparse per-region cell counts noted above, a generalized linear mixed model (binomial mixed logit with a random farm intercept, fitted by variational Bayes) was used to obtain farm-balanced, population-averaged estimates of regional seroprevalence, in which farm-level random effects are partially pooled so that heavily sampled farms do not dominate their region’s estimate. This model reproduced the extremes of the regional distribution (West Greece highest, 60.1%; Thessaly lowest, 11.0%; Table 5), but gave markedly lower estimates than the raw proportions for regions whose raw value was driven by a few intensively sampled, high-prevalence farms—most noticeably Peloponnese (*n* = 3 farms; raw 54.1% → 42.3%), Eastern Macedonia and Thrace (53.6% → 40.7%) and Central Macedonia (31.2% → 24.4%)—while leaving the best-supported regions (Epirus, *n* = 14 farms, 48.7% → 47.9%; West Greece; Thessaly) comparatively unchanged. This analysis therefore leaves the qualitative regional pattern unchanged. Model specification and estimation details are given in Section 2.5.

Figure 1 presents these raw and GLMM population-averaged regional estimates as bar charts with 95% confidence intervals, providing a visual complement to Table 4 and Table 5.

### 3.5. Between-Farm Heterogeneity

The intraclass correlation coefficient (ICC) for swIAV seropositivity across canonical farms was 0.21. Because this ICC was estimated at the farm level (the clustering unit, 44 farms), the design effect is based on the mean number of valid samples per farm (45.5; median 31.5, range 3–210, CV 0.90), which varied because several age/parity strata were sampled per report (typically 3–5, occasionally up to 20 animals per stratum) and some farms contributed several reports. Assuming equal cluster sizes, DEFF = 1 + (45.5 − 1) × 0.21 ≈ 10.4, corresponding to an effective sample size of approximately 190 rather than the nominal 2003; accounting for unequal cluster sizes (Section 2.5), DEFF ≈ 18.1 and the effective sample size is approximately 110. At the farm-year level (61 units; mean 32.8, median 30, range 3–140, CV 0.68; ICC 0.23), the corresponding design effects were 8.3 and 11.8. Because DEFF is sensitive to cluster size, these values should be regarded as approximate. This substantial inflation of variance relative to a naive assumption of independent individual observations directly motivates the use of farm-clustered GEE models—and the small-sample bias-reduced covariance estimator—in addition to conventional chi-squared tests for all comparisons reported above, and cautions against over-interpreting subgroup estimates based on few farms.

As a further robustness check on this clustering structure, we re-examined the overall seroprevalence estimate after excluding the two most intensively resampled farms in the dataset (one farm in West Greece, five sampling events; one farm in Epirus, four sampling events), which together contributed a disproportionate share of individual records relative to other farms. Overall seroprevalence was essentially unchanged (45.6%, 766/1681, versus 46.4%, 929/2003, in the full dataset), indicating that the headline estimate is not materially driven by the sampling frequency of these two farms. Because these two farms belong to West Greece and Epirus, i.e., regions at the upper end of the regional distribution, stratified estimates after their exclusion were also examined (Supplementary Table S5): seroprevalence was 71.4% (75/105; three farms) in West Greece and 50.3% (268/533; 13 farms) in Epirus, the other regions being unaffected; the regional GEE *p*-value was < 0.001 with the standard estimator and 0.13 with the bias-reduced estimator, and the age–stage pattern was essentially unchanged (stage *p* < 0.001). Thus, the high regional estimates for West Greece and Epirus were not driven by the repeated sampling of these two farms, although the West Greece estimate then rests on only three farms.

## 4. Discussion

### 4.1. Comparison with Prior Greek Surveillance

The swIAV seroprevalence reported here (46.4% overall) lies between the vaccinated- and unvaccinated-farm estimates reported in a recent Greek nationwide study using the same CIVTEST SUIS INFLUENZA ELISA kit, which found 54% seroprevalence in vaccinated and 23% in unvaccinated commercial farrow-to-finish farms sampled between 2019 and 2023 [8]. Because the two studies share authors and geographical setting, potential overlap between the datasets was examined. The study in [8] was conducted between March 2019 and April 2023, with blood samples collected specifically for that research by stratified random sampling within farms that reported no clinical signs of respiratory disease, whereas the present study analyses routine diagnostic submissions; none of the diagnostic records analysed here was included in the dataset of [8]. Some farms may have contributed to both studies; however, the exact number of overlapping farms could not be established, because the two datasets were compiled independently and for different purposes, and farm identities were not cross-referenced between them. Farm-level overlap therefore cannot be excluded and is not necessarily confined to the period of temporal overlap described below. Temporal overlap is restricted to January–April 2023, when 366 samples (151 positive) from 12 reports on 11 farms were collected in the present study; excluding these samples, overall seroprevalence was 47.5% (778/1637; farm-clustered 95% CI 40.4–54.8%), the age–stage pattern was unchanged, and the regional association remained significant under the standard estimator (*p* = 0.031) but not under the bias-reduced estimator (*p* = 0.28). The comparison with [8] also allows the direction of selection bias to be gauged: [8] used stratified random sampling in farms without clinical respiratory signs and reported 54% seroprevalence in vaccinated and 23% in unvaccinated farms, whereas the present overall estimate (46.4%) lies within this range and seroprevalence in unvaccinated growers/finishers (approximately 24%) closely matches that of the unvaccinated farms in [8]. This suggests that preferential submission of samples from farms with respiratory problems did not grossly inflate the present estimates, although differences in study period, farms and design preclude a formal assessment. Relative to [8], the present study contributes a later observation period (2023–2026), a finer regional breakdown (seven vs. three areas), a standardized six-stage age classification and cluster-adjusted analyses of temporal and regional patterns. The higher seroprevalence observed here in (vaccinated) sows relative to (unvaccinated) growers/finishers is directionally consistent with Papatsiros et al. (2023), who reported a similar age pattern in unvaccinated growing pigs (declining from 18% at 4 weeks to a nadir of 9% at 12 weeks, then rising to 22% by 20 weeks) alongside a monotonic rise in seroprevalence with sow parity (gilts 27%, parity 1–2 34%, parity 3–4 38%, parity 5–6 44%, unvaccinated farms) [8]. Because breeding animals in the present study were vaccinated whereas those in the unvaccinated arm of [8] were not, the two sow estimates are not directly comparable. The more robust cross-study concordance is the low, trough-shaped seroprevalence in unvaccinated growing pigs, which is consistent between datasets and may be compatible with a shared process of waning MDA followed by limited natural exposure during the growing period, although this interpretation cannot be confirmed with cross-sectional serological data.

### 4.2. Comparative Perspective: swIAV Seroprevalence Across Europe

The current overall estimate (46.4%) is markedly lower than the remarkably stable, very high seroprevalence reported by three sequential French national ELISA surveys—91% in 2008, 87% in 2018 and 91% in 2022 [19] (batch-level estimates in fattening pigs at slaughter, not per-animal prevalence). Herd-level estimates such as the >85% herd-level seroprevalence reported in sows from a Spanish retrospective diagnostic-laboratory survey [20] and the 60–100% (median) herd-level seroprevalence reported among Belgian fattening herds [21] are not directly comparable with our individual-level figure. An earlier four-country study spanning Belgium, France, Italy and Spain similarly documented widespread but variable herd-level swIAV circulation across intensively managed European pig farms [22]. At the opposite extreme, a 2023 Serbian survey of small-scale, low-biosecurity backyard swine holdings found 0% herd-level swIAV seroprevalence [23]. A separate, randomized Spanish farm survey (98 farms, 2151 pigs) found individual-animal seroprevalence of 62.3% (95% CI 60.2–64.3%), with 93.9% of farms having at least one seropositive pig [24], while a Croatian retrospective study reported a markedly lower individual-animal seroprevalence of 30.3% by nucleoprotein ELISA (62.2% reactive to at least one subtype by haemagglutination inhibition) [25]. This illustrates that, even when restricting comparison to individual-animal ELISA estimates, European swIAV seroprevalence ranges from below one-third to above three-fifths of pigs tested, depending on country, sampling frame and assay, underscoring how strongly seroprevalence depends on production intensity, herd size and biosecurity level rather than reflecting a single, generalizable “European” baseline. This point is reinforced by an earlier large-scale North American survey (>111,000 porcine sera tested by haemagglutination inhibition, 1998–2000) that found 22.8% overall swIAV seropositivity, confirming that comparably wide within-country variability in swIAV circulation is not unique to Europe [26]. The apparent difference relative to the French estimates may reflect several factors rather than genuinely lower Greek viral exposure: France’s much larger, denser pig population favouring near-saturating enzootic circulation of multiple co-circulating lineages; likely differences in vaccination strategy and coverage between the two national contexts, which directly shape the age/parity-related seropositivity patterns described above; differences in sampling design (the present convenience sample of diagnostic submissions versus the French surveys’ structured national sampling frame), which are not directly comparable and may not equally capture the full range of herd-level exposure; and differences in laboratory methods. Although both the French surveys and the present study used ELISA, the French surveys used a different NP ELISA (ID Screen^®^ Influenza A Nucleoprotein Swine Indirect, positive at S/P > 0.4) and, importantly, expressed seroprevalence at the batch level—a batch of 10 fattening pigs sampled at slaughter being classified as positive if at least one pig was seropositive—rather than per animal [19]. The two estimates are therefore not directly comparable, and the apparent gap is largely a consequence of the different measurement levels and assays rather than evidence of lower viral exposure in Greece. A large Italian farm-based passive surveillance programme (253 farms, 2013–2022) similarly reported a herd-level swIAV detection rate of 37.9%, reinforcing that detection-based herd prevalence estimates from structured surveillance networks (ESNIP3, Pan-European, Italy) are systematically not comparable to individual-animal seroprevalence figures such as ours [27]. These serological and virological estimates are summarized in Table 6.

### 4.3. Regional Heterogeneity

In unadjusted analyses, farm location was associated with swIAV seroprevalence under standard cluster-robust testing, but not under the bias-reduced correction (although the association persisted after adjustment for year and stage), with West Greece highest (61.0%) and Thessaly lowest (10.1%). We initially considered whether this gradient reproduced the regional heterogeneity previously reported for Greek pig farms by Papatsiros et al. [8]; on inspection, however, the two studies place Thessaly in opposite rank order. Papatsiros et al. [8] reported the highest swIAV seroprevalence among unvaccinated farms in North Greece and West Greece, together with Thessaly, whereas in the present dataset Thessaly returned the lowest regional seroprevalence of all seven regions while West Greece remained among the highest. This divergence for Thessaly most plausibly reflects the coarser three-region grouping used in [8] (North, Central and West Greece) compared with the finer seven-region breakdown adopted here, differences in the vaccination status and farm composition contributing to each region between the two studies, and the modest number of farms per region in both datasets, rather than a genuine and stable geographic pattern of viral circulation. Although the regional association persisted after adjustment for year and stage in the independence GEE model (Section 3.4), the adjusted estimates were very imprecise, age-group composition and sampling period varied across regions, and vaccination practices could only be documented at the protocol level; together with the small number of farms in several regions, these factors mean that the regional findings should be regarded as explicitly exploratory.

## 5. Practical Implications and Recommendations

The following recommendations are directions for future surveillance and research rather than conclusions supported by the present serological dataset.

- Extend molecular (PCR/ddPCR or RT-PCR) surveillance for swIAV beyond serology; antibody detection alone cannot confirm active viral circulation or distinguish vaccine-induced from infection-induced antibodies.
- Maintain and, where possible, expand subtype and lineage characterization of circulating swIAV strains to clarify whether cross-reactivity with avian-origin antigens contributes to observed regional or temporal patterns. Because the present serological data cannot reveal which subtypes are circulating, establishing their distribution and relative prevalence in Greek pig herds is a priority for future, virus-detection-based (molecular) surveillance.
- Prioritize region-specific investigation of the markedly lower swIAV seroprevalence observed in Thessaly relative to West Greece and Eastern Macedonia and Thrace, treating it as a hypothesis to be tested in a larger, regionally balanced sample, given the lack of cross-study replication, the imprecision of the adjusted estimates, and the loss of significance under the more conservative bias-reduced correction (Section 4.3).
- Where feasible, link swine and poultry/wild-bird influenza surveillance data at the regional level to support early detection of any future cross-species signal, without implying that current swIAV seroprevalence reflects avian exposure.
- Extend targeted biosecurity guidance—species separation, restricted wild-bird access, controlled water sources—to mixed-species smallholdings keeping pigs alongside backyard poultry, a setting associated elsewhere with frequent human-to-swine influenza A virus transmission [29].

## 6. Limitations

- The farm sample (*n* = 46) is a non-randomized convenience sample reflecting the client base of a single diagnostic laboratory and may not be fully representative of the Greek swine industry. In particular, because participation depended on each farm’s decision to submit samples to this specific laboratory, farms experiencing clinical respiratory disease (and hence a greater incentive to test) may be over-represented relative to healthy or non-submitting farms. This selection mechanism could bias the seroprevalence estimate and formally precludes extrapolation to the national herd. Because the reason for submission was not recorded in the available dataset, the estimates could not be stratified by clinical versus routine submissions; accordingly, the results are presented as laboratory-submission-based descriptive estimates rather than a national baseline, and comparisons with future studies based on different sampling motives should be made with caution. Comparison with a randomly sampled Greek dataset [8] suggests, however, that this bias is unlikely to be large (Section 4.1).
- Individual-animal vaccination status, exact timing, and product- or batch-level details (including the commercial vaccine brand used on each farm) were not available; consequently, swIAV seropositivity cannot be attributed to natural infection versus vaccination at the individual-animal level, particularly in routinely vaccinated breeding animals, and the upper end of the age gradient is largely vaccine-driven. In addition, no age-specific antibody titres or MDA decay data were available, so residual MDA in nursery/weaners (4–8 weeks) could not be quantified.
- The study period ended in May 2026, so 2026 represents a partial year; seasonal differences in sample collection across years and repeated sampling of some farms in different years may have influenced the temporal comparison.
- The ELISA panel used cannot distinguish antibody responses elicited by avian-, human- or classical swine-lineage influenza A viruses, and no molecular or subtyping data are presented; the mixing-vessel and avian–swine-interface material is therefore contextual and forward-looking rather than a finding of this study.
- Molecular (oral fluid) swIAV data fell outside the dataset’s blood-only inclusion criterion and could not be incorporated, thereby limiting assessment of active viral circulation and lineage identification.
- Several regions and some age strata are represented by few farms or animals; the corresponding subgroup point estimates are unstable, and the fully adjusted regional model converged only under the independence working correlation. Although the regional effect persisted after adjustment, the adjusted estimates were imprecise. A generalized linear mixed model with a random farm intercept was fitted as a descriptive complement (Section 3.4, Table 5); it reproduced the extremes of the regional distribution while giving lower estimates for regions dominated by a few intensively sampled farms, and the raw region-level proportions in Table 4 should be read alongside these farm-balanced values rather than in isolation.

## 7. Conclusions

Analysis of swIAV serological data from 44 Greek commercial pig farms (2023–2026) revealed an overall seroprevalence of 46.4%, with at least one seropositive animal in 51 of 61 farm-year sampling units (83.6%) and in 36 of 44 unique farms (81.8%), but with pronounced difference across production stages. Seroprevalence was also high in suckling piglets (70.7%, *n* = 198 from 23 farms), most plausibly reflecting maternally derived antibody from vaccinated dams; among the unvaccinated stages, seroprevalence declined from nursery/weaners to a trough in growers and finishers, and the high value in breeding sows is likely to largely reflect routine vaccination. Seropositivity in unvaccinated animals indicates prior exposure but does not by itself demonstrate active viral circulation at the time of sampling. No robust temporal trend was detected across the 2023–2026 study period, although some adjusted models suggested higher seroprevalence in 2025–2026. A regional gradient (lowest in Thessaly, highest in West Greece) was significant under standard cluster-robust testing but not under the more conservative small-sample correction in unadjusted analyses (although it persisted after adjustment for year and stage), and it was not reproduced in the same direction by an earlier Greek dataset, and rests on several regions with few farms; it is therefore reported as hypothesis-generating and requires confirmation. The overall estimate is consistent with prior Greek and European surveillance, which together illustrate that swIAV seroprevalence varies substantially with production intensity, vaccination coverage, age structure and region. Because the serological assay cannot identify influenza A subtypes or lineages, distinguish active from past infection, or establish transmission between species, future research should add molecular, subtyping-capable swIAV surveillance to serological monitoring and, given Greece’s recurrent HPAI activity, explore integrated One Health surveillance; these are directions for future work rather than conclusions demonstrated by the present data. As a laboratory-submission-based convenience sample, the present estimates are descriptive and not nationally representative.

## Figures and Tables

**Table 1:** Distribution of participating farms and total breeding sow capacity by administrative region of Greece. Farms (*n*) refers to all 46 participating farms, 44 of which contributed swIAV serology.

Region	Farms (*n*)	Total Sows
Epirus	14	6100
Thessaly	7	3650
Eastern Macedonia & Thrace	7	3450
Central Macedonia	6	3150
Central Greece	5	1820
West Greece	4	2600
Peloponnese	3	2150
**Total**	**46**	**22,920**

**Table 2:** swIAV seroprevalence by sampling year. Crude chi-squared *p* < 0.001; farm-clustered categorical GEE *p* = 0.50; farm-clustered linear-trend OR = 1.12/year (*p* = 0.29); 2026 = January–May only (partial year). Laboratory reports (unique farms) per year: 2023, 18 (16); 2024, 12 (11); 2025, 22 (21); 2026, 14 (13); 95% CIs are individual-level Wilson intervals that do not account for within-farm clustering (descriptive only); farm-clustered 95% CIs (intercept-only GEE, bias-reduced covariance estimator) are shown in parentheses; *p*-values are from farm-clustered models.

Year	*n* Positive/*n* Tested	Seroprevalence (%)	95% CI (Wilson) and Farm-Clustered GEE (in Parentheses)
2023	197/483	40.8%	36.5–45.2% (27.7–55.3%)
2024	112/270	41.5%	35.8–47.4% (26.2–58.6%)
2025	439/855	51.3%	48.0–54.7% (40.7–61.9%)
2026	181/395	45.8%	41.0–50.8% (30.6–61.8%)

**Table 3:** swIAV seroprevalence by age/production stage. Note that multiparous sows (and, on some farms, gilts) were swIAV-vaccinated; the high seroprevalence in suckling piglets is consistent with MDA, although this cannot be confirmed with cross-sectional serology (Section 4.1). Stage information was available for 1989/2003 samples (927/929 positive); 14 samples from 2023 (12 without an age label and 2 from boars; 2 positive) are excluded. The 95% CIs are individual-level Wilson intervals that do not account for within-farm clustering (descriptive only); farm-clustered 95% CIs (intercept-only GEE, bias-reduced covariance estimator) are shown in parentheses.

Age/Production Stage	*n* Positive/*n* Tested	Seroprevalence (%)	95% CI (Wilson) and Farm-Clustered GEE (in Parentheses)
Suckling piglets (≤3 wk)	140/198	70.7%	64.0–76.6% (57.8–80.9%)
Nursery/weaners (4–8 wk)	204/529	38.6%	34.5–42.8% (29.6–48.4%)
Growers (9–16 wk)	114/460	24.8%	21.1–28.9% (16.9–34.8%)
Finishers (> 16 wk)	42/175	24.0%	18.3–30.8% (12.8–40.5%)
Gilts/nulliparous sows	75/163	46.0%	38.5–53.7% (34.5–58.0%)
Multiparous sows (vaccinated)	352/464	75.9%	71.8–79.5% (63.5–85.0%)

**Table 4:** swIAV seroprevalence by administrative region. Regions are listed for descriptive purposes only; the order should not be interpreted as a ranking of true regional differences. † Regions represented by fewer than 5 farms, whose estimates are particularly unstable. A sensitivity analysis merging them with adjacent regions is given in Supplementary Table S7. Farms (*n*) refers to all participating farms; farms contributing swIAV results were 4 in Central Greece and 6 in Eastern Macedonia and Thrace (unchanged elsewhere). The 95% CIs are individual-level Wilson intervals that do not account for within-farm clustering (descriptive only); farm-clustered 95% CIs (intercept-only GEE, bias-reduced covariance estimator) are shown in parentheses. Crude *p* < 0.001; farm-clustered standard robust FDR *p* = 0.014; bias-reduced FDR *p* = 0.39.

Region	Farms (*n*)	*n* Positive/*n* Tested	Seroprevalence (%)	95% CI (Wilson) and Farm-Clustered GEE (in Parentheses)
West Greece †	4	192/315	61.0%	55.5–66.2% (37.9–79.9%)
Peloponnese †	3	133/246	54.1%	47.8–60.2% (11.3–91.5%)
Eastern Macedonia & Thrace	7	155/289	53.6%	47.9–59.3% (38.2–68.4%)
Epirus	14	314/645	48.7%	44.8–52.5% (39.7–57.8%)
Central Greece	5	44/129	34.1%	26.5–42.6% (12.3–65.6%)
Central Macedonia	6	78/250	31.2%	25.8–37.2% (10.5–63.7%)
Thessaly	7	13/129	10.1%	6.0–16.5% (1.4–46.1%)

**Table 5:** GLMM (binomial mixed logit with region as fixed effect and random farm intercept, variational Bayes; Python statsmodels 0.15) population-averaged regional seroprevalence estimates, alongside raw proportions for comparison. Differences from the raw values are largest for regions whose raw estimate was dominated by a few intensively sampled farms. Values recalculated from the full dataset. Descriptive display only; not used for inference.

Region	Raw Seroprevalence (%)	GLMM Population-Averaged (%)	95% Credible Interval
West Greece	61.0%	60.1%	55.9–64.1%
Eastern Macedonia & Thrace	53.6%	40.7%	36.3–45.3%
Epirus	48.7%	47.9%	44.9–50.9%
Peloponnese	54.1%	42.3%	37.1–47.7%
Central Greece	34.1%	34.2%	27.8–41.2%
Central Macedonia	31.2%	24.4%	20.5–28.8%
Thessaly	10.1%	11.0%	6.6–17.3%

**Table 6:** swIAV seroprevalence in the present study compared with selected European surveys, with the measurement level (individual-animal vs. herd) shown for each estimate. Herd-level and individual-level prevalences are not directly comparable and are labelled accordingly; the Serbian figure derives from low-biosecurity backyard holdings. Sampling period and diagnostic test are shown for each survey were reported in the source publication; “not reported” indicates that the primary source did not state an exact sampling period. NP-ELISA, nucleoprotein ELISA. Estimates based on antibody detection (seroprevalence) and on virus detection (RT-PCR, virus isolation or sequencing) measure different outcomes and must not be compared numerically; virus-detection studies are shown for context only. To make this distinction explicit, the table is divided into two sections: (**A**) serological studies reporting antibody prevalence, and (**B**) molecular/virological studies reporting virus detection, which are shown for context only and are not directly comparable with the seroprevalence estimates in section A.

Country/Survey	Level	Estimate	Period	Test Method	Ref.
**A. Serological studies—antibody prevalence (ELISA/HI)**					
Present study (Greece)	Individual	46.4%	2023–2026	ELISA (NP; CIVTEST SUIS INFLUENZA)	—
France (national)	Batch (10 fattening pigs; ≥1 positive)	87–91%	2008, 2018, 2022	NP-ELISA (ID Screen; S/P > 0.4)	[19]
Spain (sows)	Herd	>85%	2003–2005	ELISA	[20]
Belgium (fattening)	Herd	60–100% (median)	not reported	ELISA	[21]
Serbia (backyard)	Herd	0%	March–August 2023	ELISA	[23]
Spain (randomized)	Individual	62.3%	not reported	NP-ELISA + HI	[24]
Croatia	Individual	30.3% (NP-ELISA)	2011–2016	NP-ELISA + HI	[25]
**B. Molecular/virological studies—virus detection (RT-PCR, virus isolation, sequencing); shown for context only, not comparable with section A**					
ESNIP3 (17 countries)	Herd (virus detection)	31%	2010–2013	RT-PCR/virus isolation	[7]
Italy (253 farms)	Herd (virus detection)	37.9%	2013–2022	Passive surveillance (RT-PCR + HI)	[27]
Pan-European (2015–2018)	Herd (virus detection)	56.6%	2015–2018	RT-qPCR/sequencing	[28]

## Data Availability

The data presented in this study are available on request from the corresponding author due to privacy and ethical restrictions.

## Supplementary Materials

The following supporting information can be downloaded at https://www.mdpi.com/article/10.3390/vetsci13101041/s1: Table S1: Number of valid swIAV serum samples by sampling year and month of laboratory reception (2026 = January–May only); Table S2: Distribution of valid samples by region and sampling year, with crude seroprevalence (S2a), and by region and age/production stage (S2b); Table S3: Raw and Benjamini–Hochberg FDR-adjusted *p*-values of the farm-clustered GEE joint Wald tests for year, stage and region, under the standard and bias-reduced covariance estimators; Table S4: All 15 pairwise age/production-stage contrasts (odds ratios, 95% CIs, raw and FDR-adjusted *p*-values); Table S5: Sensitivity analysis excluding the two most intensively resampled farms: regional (S5a) and age/production-stage (S5b) estimates; Table S6: Multivariable models for swIAV seropositivity (full and reduced GEE with independence working correlation, full GEE with exchangeable working correlation, and Firth penalized logistic regression); Table S7: Sensitivity analysis with regions represented by fewer than 5 farms merged with a geographically adjacent region.

## Abbreviations

The following abbreviations are used in this manuscript:

swIAV	Swine influenza A virus
ELISA	Enzyme-linked immunosorbent assay
NP	Nucleoprotein
MDA	Maternally derived antibody
PRDC	Porcine respiratory disease complex
PRRSV	Porcine reproductive and respiratory syndrome virus
HPAI	Highly pathogenic avian influenza
ESNIP	European Surveillance Network for Influenza in Pigs
GEE	Generalized estimating equation
GLMM	Generalized linear mixed model
ICC	Intraclass correlation coefficient
DEFF	Design effect
FDR	False discovery rate
OR	Odds ratio
CI	Confidence interval
QIC	Quasi-likelihood under the independence model criterion
RT-PCR	Reverse transcription polymerase chain reaction
